# Linking up New Hospital Units

**Published:** 1914-08-15

**Authors:** 


### The Hospital

The Workers' Newspaper of Administrative Medicine and Institutional Life, Administration, National Insurance and Health.

No. 1469, Vol. LVI. SATURDAY, AUGUST 15, 1914. PRICE ONE PENNY.

### LINKING UP NEW HOSPITAL UNITS.

Almost in a moment, without any previous preparation or apprehension, the inhabitants of the United Kingdom have been called to war in defence of causes the upholding of which has made England great and created the British Empire. The strain upon the Government and many of the great departments of the State has become enormous, and they properly look to civilians of all ranks and classes, according to capacity, to unite heart and soul to uphold and defend the right. Our special interest is necessarily with the provision which will be required and must be made in advance to secure to the utmost extent every facility which will tend to diminish suffering and restore to health and active service those engaged in the Army and Navy.

Lord Kitchener's appeal is calling forth a splendid response which does credit to the manhood of every nationality represented in the United Kingdom. It proves, we are thankful to know, that this war will be an effective remedy for the threatened loss of moral force with which the nation was menaced. We are all justifiably proud of our Navy, and we hope that the shortage of naval doctors will speedily cease to be a factor in the present anxious position of public affairs. Every resident in these islands feels deeply that nothing must be left undone to encourage our Navy and Army by making the most efficient and full provision for their interests arising from service on sea and land. We hope that the casualties caused by war in the case of our own defenders may mercifully prove relatively few; but the magnitude of the forces engaged, the enormity of the weapons of destruction that will be employed, and the consequent immensity and seriousness of the risks which we have to incur, must make the civilian population, as just and proud men, provide adequate funds with prodigal liberality; so alone will it be possible to secure that, so far as human knowledge and forethought can provide, no single thing will be wanted to diminish suffering and to expedite recovery.

The position to be faced is not free from difficulty. To meet it effectively will require co-operation of the closest kind, resting upon mutual confidence between the civilian, naval and military authorities. The policy of our great voluntary hospitals and their first duty will be to secure adequate care and treatment for non-combatant members resident in these islands. A state of war will necessarily tend to increase the demands upon our hospitals, especially from the poorer members of the community. Those demands are likely to increase rather than to diminish as the war continues, and it is, consequently, of the first importance that the managers of every one of our voluntary hospitals should carefully forecast and faithfully provide the means to meet any increased demand for in-patient treatment, especially through the causes just mentioned.

Our voluntary hospitals will, further, have not only to spare members of their visiting medical and resident staffs, but to supply, as they have already done, a number of fully trained and certificated nurses of the highest character and ability; and, in the case of clinical hospitals, to encourage the senior and most capable students to act as dressers under the control of the naval and military authorities. The clinical hospital managers will, further, have to co-operate with the universities, schools and colleges with the object of keeping up the supply of doctors and nurses especially, so as to enable our defenders of all ranks to be adequately cared for as necessity may arise. It is well that the public should appreciate these grave facts, for they prove that the first duty of every thoughtful resident throughout the country is to remember the voluntary hospitals, and in forecasting the economical arrangements of the household to send at once some gift, however small, to the nearest voluntary hospital in their district.

The Government no doubt will make a large grant towards the cost of equipping and maintaining the new units rendered possible by the liberality of many men and women, but the success and usefulness of all these new establishments for the reception of the sick and wounded must depend upon a perfected system of organisation.

The medical departments of the Army and Navy will have their hands full quite apart from the direction and control of the emergency hospitals, which have to be organised and equipped largely with the co-operation and aid of the citizens. In our view it would be a prudent and fruitful source of strength if Lord Kitchener were to add to his staff a Civil Director-General of proved knowledge and administrative capacity, whom he could make responsible for the selection and equipment of the supplementary units for the reception of the sick and wounded and of Service convalescents throughout the country. He could not fail to find this step of great importance, for it would remove many difficulties and expedite the gathering up of all the means to hand for supplying the requisite hospital and convalescent accommodation and making it rapidly available, sufficient, and complete. People are offering houses, buildings, golf-club pavilions, schools, and a multitude of other buildings, some of them relatively small and few of them quite ideal, which will require to be handled and judged with a fullness of knowledge, in order that every one of them which can be made effective for its purpose should be promptly accepted and made ready for occupation. **Meanwhile every thoughtful man and woman throughout the United Kingdom should make a point of sending at once some contribution to the nearest voluntary hospital without a moment's delay.**

